# Enhancing neonatal resuscitation outcomes: bridging theory and practice

**DOI:** 10.1007/s00431-025-06087-8

**Published:** 2025-03-19

**Authors:** Pankaj Soni, Manjunath Mallikarjuna Nagalli

**Affiliations:** 1 Department of Neonatology, Thumbay University Hospital, Ajman, UAE; 2 Department of Clinical Sciences (Pediatric Neonatology), College of Medicine, Gulf Medical University, Ajman, UAE; 3 Department of Pediatrics, Burjeel Speciality Hospital, Sharjah, UAE

**Keywords:** Airway management, Clinical trials, Global variation, Guidelines, Low-resource settings, Neonatal resuscitation program, Neonatal care, Medical advances

## Abstract

Neonatal resuscitation practices have undergone tremendous changes over the past two decades, with progress accelerating due to advances in medical technology, scientific research, and improvements in clinical practices. Among other global frameworks, the neonatal resuscitation program has been periodically updated to incorporate evidence-based interventions aimed at improving survival rates, reducing morbidity and enhancing long-term health outcomes for newborns. This review traces the historical development of neonatal resuscitation guidelines from the early days’ resuscitation practices of the mid-twentieth century to the present day. It narrates how clinical needs, emerging technologies, and scientific discoveries have shaped the evolution of these guidelines and practices. By reviewing recent guidelines, such as those issued by the American Heart Association and the World Health Organization, this article sheds light on the current core principles of neonatal resuscitation, including effective airway management, appropriate ventilation techniques, and the critical importance of timely intervention. The major determinants of changes in guidelines, as identified through this review, include advancements in scientific research, expert opinion, and international collaboration. Challenges in implementing these guidelines, particularly in low-resource settings, are discussed, along with case studies that demonstrate the impact of updated practices in real-world clinical environments.

*Conclusion*: The review concludes with a reflection on the continued need for research to close remaining gaps and enhance neonatal resuscitation practices across diverse global contexts.

**Table:** 

**What is Known:**
• *Effective neonatal resuscitation significantly improves outcomes by reducing neonatal mortality and morbidity.*
• *Neonatal resuscitation program provides standardized protocols to implement these resuscitation skills.*
**What is New:**
• *Simulation-based training and real-time feedback can bridge the gap between theoretical guidelines and practical application.*
• *Despite advances in knowledge and application of these guidelines, challenges persist, such as regional differences, lack of resources in lower-income countries, and ideal ventilation devices and oxygenation methods.*

## Introduction

Neonatal resuscitation is an emergency procedure aimed at stabilizing newborns who are unable to breathe independently at birth and supporting ventilation once breathing is established. Globally, birth asphyxia accounts for more than 800,000 neonatal deaths annually, with a disproportionately higher burden observed in low- and middle-income countries (LMICs) [1]. Standardized guidelines and training programs are crucial in reducing these deaths through timely and effective resuscitation. Neonatal resuscitation protocols for airway clearance, ventilation, and chest compressions have been developed based on evidence and are continuously updated with advancements in neonatal science and technology [2].

The field of neonatal resuscitation has undergone significant transformation over the past decades, driven by continuous research and international collaboration. Most simplistic and ad hoc practices are being replaced with evidence-based protocols developed through rigorous systematic literature reviews conducted by the International Liaison Committee on Resuscitation (ILCOR). For example, the American Heart Association (AHA) published a comprehensive update in 2020, incorporating education and post-resuscitation care and strategies relevant to resource-limited environments [2]. Similarly, ILCOR and the American Academy of Pediatrics (AAP) released their updates in 2023, focusing on optimally delaying cord clamping (DCC) and the use of positive pressure ventilation (PPV) [3, 4]. The European Resuscitation Council (ERC) has started developing resuscitation guidelines for 2025, which will be released by the end of 2025. These updates underscore an evolving commitment to refining techniques that improve neonatal survival and minimize long-term complications.

Advanced technologies have also contributed to the evolution of neonatal resuscitation guidelines. For instance, feedback ventilation devices that give real-time feedback have been shown to increase compliance with resuscitation algorithms, thereby lowering the likelihood of human error [5]. This narrative review seeks to trace and explore the historical evolution and current landscape of neonatal resuscitation guidelines and provide a comprehensive overview of evidence-based practices in their development and implementation globally. We identified some of the gaps and trends in neonatal resuscitation practices by integrating data from recent updates, including the updates released by AHA in 2020 and 2023, as well as ILCOR recommendations. The implications of these guidelines on clinical practice have been emphasized, particularly in diverse healthcare settings, along with the role of emerging technologies in improving neonatal outcomes.

Moreover, this review explores the challenges associated with guideline implementation in resource-limited settings, thus creating space for innovation and collaboration. By highlighting these challenges, the review offers actionable insights for improving global neonatal resuscitation efforts and ensuring equitable access to life-saving interventions for all newborns, regardless of their geographic or socio-economic context. In conclusion, this review aims to contribute to ongoing efforts to optimize neonatal care in reducing global disparities and improving outcomes for vulnerable newborn populations [3, 5].

## Evolution of neonatal resuscitation methods

### Early resuscitation methods

Neonatal resuscitation has its roots in primitive and rudimentary practices, reflecting limitations in the scope of knowledge, especially the availability of medical interventions. Historical intervention techniques employed after failed initiation of respiration in the neonate began with simple physical stimulation and evolved into gross artificial attempts of respiration. At the beginning of the twentieth century, midwives and physicians generally relied on practices such as slapping the newborn’s back, applying cold water, and using basic suction devices to clear the airways [6]. Although these approaches were intended to be beneficial, they often lacked scientific evidence and resulted in more harm than good in some cases. The high rates of neonatal mortality in this period established the requirement for systematic developments in neonatal care.

A landmark change in mid-20th-century medicine was the acknowledgement by physicians of the importance of ventilation support for neonates. The development of PPV in the 1950s revolutionized neonatal resuscitation, as it offered an effective approach for supporting the breathing of asphyxiated newborns [7] (Table 1). However, there were no established standards, which led to considerable variability among practitioners. This inconsistency highlighted the urgent need for evidence-based guidelines.

**Table 1:** Evolution of neonatal resuscitation practices

Year	Event/development	Details
**1950s**	Introduction of positive pressure ventilation (PPV)	Acknowledgment of the importance of ventilation support for neonates. PPV allowed effective breathing support for newborns with asphyxia
**1953**	Introduction of the Apgar score	Dr. Virginia Apgar developed a standardized measure to assess neonate health at birth, which became a cornerstone of neonatal resuscitation
**1970s**	First official guidelines	The AAP and AHA recognized the need for standardized protocols. The guidelines focused on airway clearance, tactile stimulation, and oxygen provision
**1980s**	Emergence of pulse oximetry	Pulse oximetry enabled non-invasive monitoring of oxygen saturation in neonates, improving oxygen therapy titration during resuscitation
**1990s**	Surfactant therapy introduced	Revolutionized care for respiratory distress syndrome in preterm infants, drastically improving survival rates
**2000s**	Advances in simulation training	High-fidelity simulations improved neonatal resuscitation training, enhancing healthcare professionals’ adherence to guidelines
**2010s**	Delayed cord clamping emphasized Heart rate monitoring	Evidence highlighted reduced intraventricular hemorrhage in preterm infants, making it a key neonatal resuscitation practice Gradual shift in methods of heart monitoring from auscultation to use of pulse oximetry and ECG
**2020**	Updated neonatal resuscitation guidelines	Reflecting medical advancements, the updated Neonatal Resuscitation Program emphasized personalized care, harm minimization, and evidence-based practices

### Development of guidelines

Neonatal resuscitation guidelines, which emerged in the last quarter of the twentieth century, represent a major milestone in pediatric care. The AAP and AHA recognized the need for standardized protocols during the 1960s and 1970s. Early guidelines were mainly based on basic interventions, such as airway clearance, tactile stimulation, and the provision of oxygen [8]. These foundational practices, though supported by limited clinical trials and consensus of specialists, led to a more methodical approach to neonatal resuscitation.

The first official guidelines on neonatal resuscitation were issued in the 1970s, marking a growing interest in improving neonatal outcomes. These guidelines introduced the critical concepts of Apgar scoring, a standardized measure developed to assess and describe an infant’s condition immediately after birth [9]. The Apgar score is considered a cornerstone of neonatal care, providing critical information about the effectiveness of resuscitative attempts and guiding further interventions (Table 1).

Updating the guidelines on neonatal resuscitation was driven by a growing evidence base and technological advancements. For instance, pulse oximetry, which utilizes an external non-invasive device, began to be used in the 1980s to monitor oxygen saturation, thereby improving the titration of oxygen therapy [6]. The continuous advances in scientific knowledge necessitate regular updates to guidelines.

### Impact of medical advances

The neonatal resuscitation guidelines were influenced considerably by medical innovations. The progress in neonatal physiology, pharmacology, and related technologies have broadened the scope of resuscitation interventions to a certain extent. One such development is the improvement made in neonatal ventilators, which have made the delivery of PPV much safer from the standpoint of barotrauma and other complications [7]. The exogenous surfactant therapy itself was first introduced as late as the 1990s and by that time had revolutionized treatment for respiratory distress syndrome, one of the most common disorders in preterm infants.

Further advancements were made by integrating simulation training within neonatal resuscitation programs. High-fidelity simulations provided an opportunity for healthcare professionals to practice and hone their skills in a controlled environment, leading to better preparedness and greater confidence to act in real-life emergencies [10] (Table 1). Such programs have been proven to improve adherence to guidelines that translate to better neonatal outcomes; thus, education must be focused on developing resuscitative care.

The recently revised neonatal resuscitation guidelines emphasize a focus on individualized care and minimization of risk. One of the examples is the introduction of delayed cord clamping (DCC) that was introduced as a major aspect of the Neonatal Resuscitation Program (NRP®) updated in 2020 (Table 1). This intervention has been shown to improve transitional circulation while reducing the incidence of intraventricular hemorrhage in preterm infants [11]. Umbilical cord milking (UCM) has been proposed as a substitute for DCC, where the umbilical cord is squeezed to deliver blood to a baby. It potentially improves the baby’s blood volume and iron levels and reduces the need for blood transfusion; however, it may cause fluctuations in cerebral blood flow and increase the chances of intraventricular hemorrhage (IVH) [12, 13]. Such measures are part of a much larger trend in pediatrics, emphasizing the possibility of optimizing the outcome for every newborn through personalized and evidence-based practice. Recent ILCOR and ERC guidelines recommend using ECG for a rapid and accurate heart rate estimation in infants requiring resuscitation [6–8].

Besides that, neonatal resuscitation recommendations have also been influenced by global health initiatives. For instance, WHO and UNICEF have continued to provide simplified, evidence-based protocols to resource-poor settings, aiming to reduce disparities in newborn care [14]. This further broadens the scope of equity and accessibility in improving neonatal resuscitation practices worldwide. The continuous refinement and adaptation of resuscitation guidelines will transform them from primitive and crude practice areas to advanced, evidence-based protocols. This reflects the dynamism of medical science and commitment towards improving neonatal outcomes. Advances in neonatology will remain in tune with innovative technologies and global health perspectives, which are essential for shaping the future of neonatal resuscitation.

## Review of current guidelines

### Neonatal resuscitation program

**Fig. 1:**
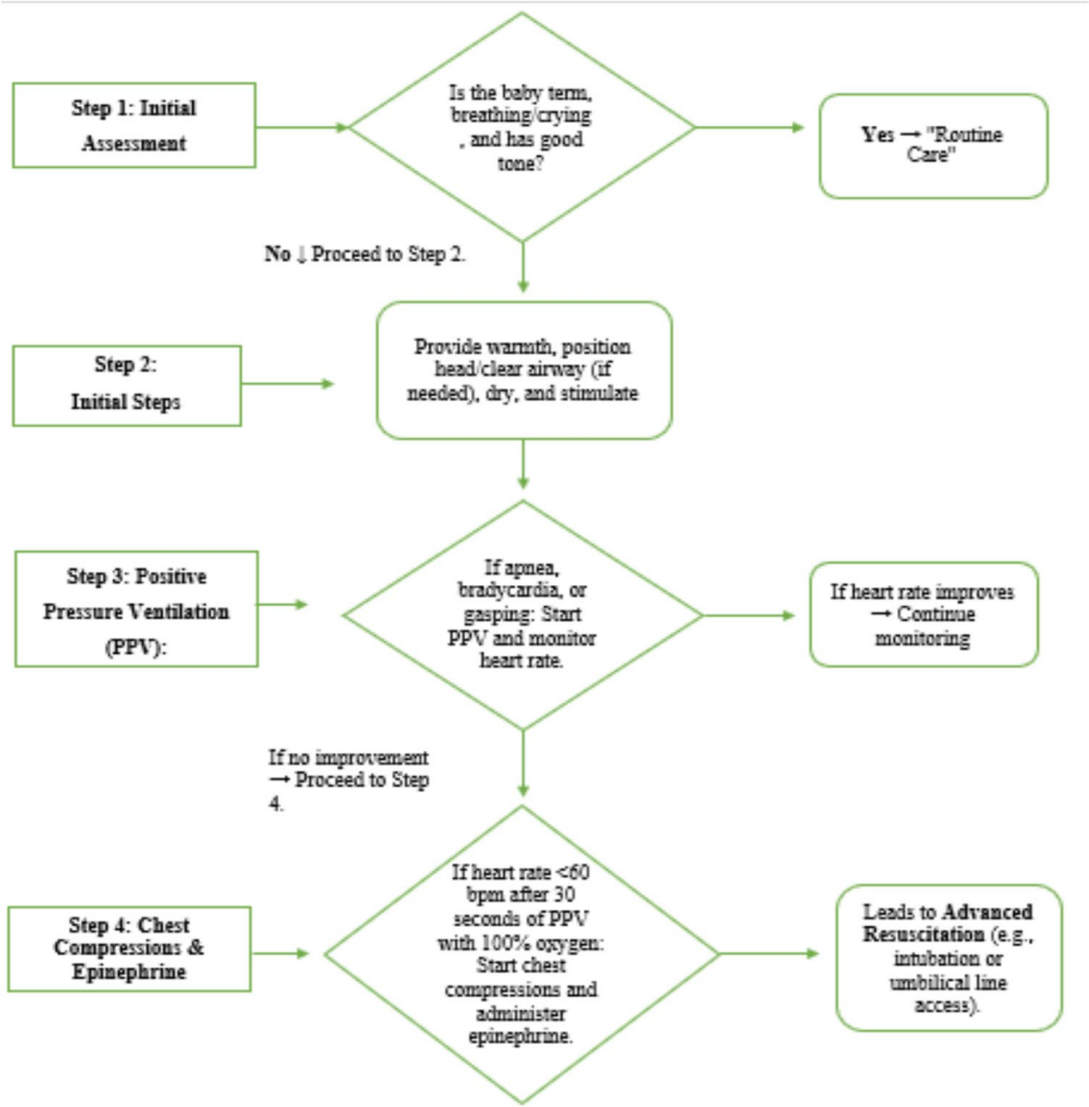
Neonatal Resuscitation Program (NRP) steps (source: American Academy of Pediatrics and American Heart Association)

Presently, NRP is widely used by most countries for neonatal resuscitation protocols. It was established by the AAP and AHA to ensure standardized care for newborns requiring immediate resuscitation after birth [8]. The latest NRP guidelines emphasize the need for a consistent assessment and management strategy for neonates in that critical first minute of life. It is based on the golden minute concept and emphasizes early assessment and interventions, such as airway clearing, stimulation, and initiation of ventilation where necessary within the first minute of life [4]. Figure 1 is a detailed flowchart that outlines the steps of the neonatal resuscitation process according to NRP guidelines; the procedure starts from antenatal briefings, sets the stage for birth assessment at term and breathing, and continues with interventions such as PPV, chest compressions, and administration of epinephrine in cases of continued bradycardia or asystole [15].

Key components of the NRP include the following:

- Initial assessment: establishing term status, tone, breathing, and heart rate of the newborn.
- Basic interventions: airway clearance, drying, warming, and stimulation.
- Advanced interventions: giving PPV, observing oxygen saturation (SpO_2_). Chest compression is done, and epinephrine is administered if bradycardia persists.
- Oxygen management: oxygen saturation targets (preductal SpO_2_) are set every minute after birth to prevent hyperoxia [16].

### International variations

Although the NRP provides the foundation, neonatal resuscitation protocols are highly different across regions. The main reasons behind this are differences in available resources, healthcare infrastructures, and regional priorities.

- American Heart Association: It follows evidence-based algorithms that align to the NRP framework and emphasizes early interventions with ventilation, which is critical for optimal results [13].
- World Health Organization: The guidelines of WHO are oriented toward resource-limited environments with a focus on fundamental interventions like clearing, drying, and stimulation of the airway and reducing the demand for sophisticated equipment [17]. According to WHO, room air should be used for resuscitation unless supplementary oxygen is required.
- European Resuscitation Council (ERC): ERC recommendations are aligned with the NRP but also place more significance on thermal management, DCC, and monitoring preductal SpO₂ in real time [8].

Though differences exist across major international guidelines, the general principle is the same—ventilation and cardiovascular support. The application of guidelines differs in providing oxygen, DCC, and resource use [18]. High-income countries like the USA and European countries are equipped with more sophisticated medical equipment, such as ECG monitoring systems, oxygen blending equipment, and pulse oximetry as standard use. Conversely, low-resource settings, such as those in sub-Saharan Africa and parts of South Asia, often face challenges in getting access to basic resuscitation tools, such as bag-mask ventilation and clean delivery practices [19].

### Evidence-based practices in neonatal resuscitation

The current neonatal resuscitation guidelines have been informed by solid evidence from clinical trials and observational studies. These studies form the basis for critical interventions, such as oxygen management, ventilation strategy, DCC, and thermal care; these all aim to help in improving neonatal survival and outcomes.

#### Oxygen management

Extensive research has been conducted on the use of oxygen in neonatal resuscitation. Traditionally, resuscitation was performed with 100% oxygen; however, further research established that this could cause oxidative stress and increased morbidity. The targeted oxygen in the resuscitation of preterm infants and their developmental outcomes (TO2RPIDO) trial (2019) correlated the much-popularized resuscitation approach using room air (21% oxygen) with significantly lower oxidative stress and better survival outcomes (Table 2). This hallmark study became a key reference for highlighting the hyperoxia-induced damage of neonatal tissue, particularly in preterm infants [20]. Thus, the guidelines released by the AHA and WHO now recommend beginning the resuscitation with room air and titrating for supplemental oxygen based on SpO₂ levels. The preductal oxygen saturation values will be determined within the first 10 min of life to make sure that optimal oxygen is present while minimizing the risk of injury.

**Table 2:** Summary of evidence-based changes in practice

Practice/intervention	Previous approach	Evidence supporting change	Current approach
Oxygen Use	100% oxygen for resuscitation	TO2RPIDO trial (2019): room air reduced oxidative stress and improved survival outcomes [20]	Use room air (21% oxygen), titrated based on SpO₂
Cord clamping	Immediate cord clamping	Delayed clamping improved hemoglobin and reduced the need for transfusions [16]	Delay cord clamping by 30–60 s unless emergency resuscitation is required
Umbilical cord milking (UCM)	Not preferred	Intact UCM was beneficial than early cord clamping in non-vigorous term and late preterm infants (> 34 weeks) and in preterm infants < 34 weeks’ gestation who do not require resuscitation	Intact UCM can be used in non-vigorous term and late preterm infants (> 34 weeks) and in preterm infants < 34 weeks’ gestation who do not require resuscitation and where DCC cannot be performed
Positive pressure ventilation (PPV)	Delayed initiation or poor ventilation techniques	Early PPV prevents hypoxia, reduces mortality, and lowers the need for escalated interventions [22]	Early initiation of PPV with corrective measures (e.g., T-piece or bag-mask ventilation)
Temperature management	Minimal focus on thermal regulation	Hypothermia is associated with increased mortality and morbidity in neonates [25]	Maintain normothermia (36.5–37.5°C) using interventions like pre-warmed delivery rooms, drying, and skin-to-skin contact

#### Delayed cord clamping and umbilical cord milking

Delayed cord clamping is another evidence-based practice that has transformed neonatal resuscitation. Traditionally, umbilical cords were clamped soon after delivery, especially in cases of emergency delivery. However, studies have shown that delaying umbilical cord clamping by 30 to 60 s increases hemoglobin values, decreases the need for blood transfusions, and reduces intraventricular hemorrhage in preterm infants [21] (Table 2). These advantages of DCC are attributed to additional placental blood that is transferred due to delayed clamping, increasing the neonate’s blood volume and iron levels. Both AHA and ERC guidelines recommend DCC in stable preterm and term neonates, provided that the neonate does not need immediate resuscitation (Table 2). When DCC cannot be performed, UCM has been proposed as a substitute, where the umbilical cord is squeezed to deliver blood to a baby. The intact-UCM involves milking the cord while it is still attached to the placenta, whereas in the cut-UCM method, the cord is first clamped and cut, and the remaining segment attached to the baby is milked. The cut-UCM approach is less common and may be used in babies where immediate clamping and cutting are necessary to start resuscitation. The UCM potentially improves the baby’s blood volume and iron levels and reduces the need for blood transfusion in preterm infants. However, it may cause fluctuations in cerebral blood flow and increase the chances of IVH. Also, limited data is available on long-term neurodevelopmental outcomes; therefore, further research is required to assess its safety and long-term safety [12, 13]. Both AHA guidelines recommend the possible use of intact UCM in non-vigorous term and late preterm infants (> 34 weeks) and in preterm infants < 34 weeks’ gestation who do not require resuscitation and where DCC cannot be performed. Intact UCM is not recommended in preterm babies < 28 weeks of gestation [7].

#### Positive pressure ventilation

Positive pressure ventilation is still the mainstay of neonatal resuscitation in the case of apnea, bradycardia, or ineffective respiratory effort among newborns. Good ventilation is essential because most cases of neonatal resuscitation are caused by respiratory insufficiency and not by cardiac arrest. Manley et al. demonstrated that early initiation of PPV is crucial, as a delay might result in hypoxia and the need for additional interventions such as chest compression or epinephrine administration [22] (Table 2). Early and effective PPV has resulted in a reduction of mortality rates and diminished the need for care escalation. The current guidelines encompass corrective measures for ventilation and respective devices, including T-piece resuscitators and bag-mask ventilation, which may be necessary for lung inflation.

#### Heart rate monitoring

Auscultation by stethoscope and palpation for a pulse at the base of the umbilical cord or the brachial or femoral arteries are simple and inexpensive methods to assess the heart rate (HR) in the delivery room, especially in low-risk newborns when the heart rate is > 100/min. However, these can be inaccurate with a tendency to underestimate the ECG or pulse oximetry values in babies needing resuscitation. Continuous monitoring by pulse oximeter (connected to the right hand) is more useful for a dynamic assessment of HR change during resuscitation; however, the initial values may underestimate the ECG a little, especially when signal quality is poor. Cold extremities, hypoperfusion, motion artifacts, arrhythmias, and ambient lighting can interfere with pulse oximetry readings. ECG can rapidly, reliably, and accurately determine the HR a few seconds earlier than pulse oximetry, especially in the first few minutes of birth. Plethysmography and Doppler are newer methods utilized for rapid and reliable HR determination but have not yet been validated in clinical trials [23, 24].

#### Temperature management

During neonatal resuscitation, normothermia must be maintained between 36.5 and 37.5°C since hypothermia is linked with increased newborn mortality and morbidity rates. A meta-analysis by Kariuki et al. showed the adverse outcomes of hypothermia associated with acute respiratory distress and metabolic instability in preterm and term infants [25] (Table 2). Evidence supports many interventions such as immediate drying, warm blankets, pre-warmed delivery rooms, and skin-to-skin contact to maintain thermal stability. The WHO and AHA guidelines also emphasize the need for these interventions to prevent heat loss, especially in preterm neonates, who are at greater risk.

Oxygen titration, DCC, earlier initiation of PPV, and thermal regulation are some evidence-based practices that have significantly impacted neonatal resuscitation outcomes. Such interventions based on robust clinical studies have a foundation in current international guidelines to ensure that newborns receive the best care possible immediately after birth.

## Key factors influencing guideline evolution

Advances in scientific research, innovation in technology, and cooperation among experts and international entities have driven the development of neonatal resuscitation guidelines. This trend ensures that the practices relating to neonatal care evolve based on the dynamic evidence base and changing clinical needs.

### Scientific research

**Fig. 2:**
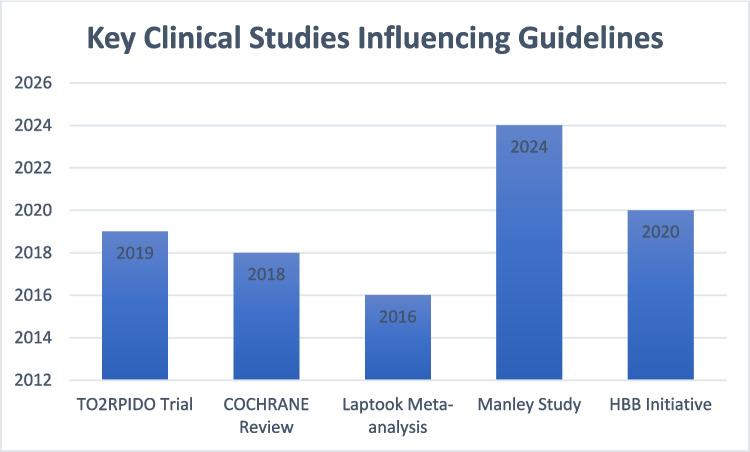
Key clinical studies influencing guidelines

Scientific research has significantly impacted the process of updating neonatal resuscitation algorithms (Fig. 2). The information gained from randomized clinical trials and systematic reviews has formed a solid foundation for practice guidance. The TO2RPIDO study showed that starting with room air and titrating the oxygen based on SpO₂ levels is better than the immediate use of 100% oxygen. An evidence-based approach in the golden hour (first 60 min) is crucial for extremely premature and extremely low birth weight neonates in terms of long-term outcomes [26]. This practice drastically decreases oxidative stress and increases neonatal survival and has been incorporated into the 2020 AHA guidelines. Findings from a study by Dodampahala et al. shed more light on the timing of DCC as it optimizes hemoglobin levels and prevents unnecessary transfusions, as well as improves cardiovascular stability among preterm infants [27]. These developments underscore the need to utilize empirical evidence to improve neonatal care.

Technological advancements have similarly transformed the practice of neonatal resuscitation by enhancing precision, safety, and effectiveness of interventions by quality initiatives (QI) [28, 29]. One of the prominent examples is the use of T-piece resuscitators and nasal continuous positive airway pressure (CPAP) for more effective delivery of PPV. Research has shown that these instruments not only reduce the rate of lung injury but are also known to improve survival rates among preterm infants. Besides all this, innovations in near-infrared spectroscopy (NIRS) are useful in measuring cerebral tissue oxygen saturation [30]. A multi-center, phase III COSGOD (cerebral oxygen saturation to guide oxygen delivery) trial investigated the role of NIRS in improving survival and reducing brain injury by monitoring brain oxygen levels in 655 preterm newborns. Survival without brain injury was slightly higher in the NIRS group (82.9% vs. 78.5%), but the difference was not statistically significant [31]. Pre-warming rooms for delivery, drying a newborn immediately after birth, and maintaining skin-to-skin contact have helped minimize complications arising out of hypothermia. These practices are aligned to the recommendations made by WHO and AHA, emphasizing neonatal body temperature being set within the 36.5–37.5°C range.

In preterm babies with respiratory distress syndrome (RDS), non-invasive high-frequency oscillatory ventilation (n HFOV) was found better in comparison to bilevel positive airways pressure (BiPAP) in terms of outcome of hospital stay, and the air leakage syndrome is used at the initial step of management [32]. Frequently, neonatal resuscitation protocols result from a consensus between pediatricians, neonatologists, researchers, and international health agencies. Hence, such initiatives offer a turning point regarding evidence-based guidelines for different healthcare settings globally through the AHA, WHO, and ERC. For instance, WHO’s attention to simplifying and cutting costs has made evidence-based neonatal resuscitation available in low- and middle-income countries. This has helped reduce disparities in health care and save lives for the less fortunate in society [33].

The evolution of guidelines for neonatal resuscitation has been made possible through a fine blend and interplay of scientific discovery, technological innovation, and global partnerships. Research forms the basis for evidence-based interventions, and its implementation has been supported by medical technological innovation. Global cooperation has also regularized the geographical scope of these guidelines to encompass the entire world and maintain equity. More investment in research and international cooperation will continue to tackle current problems and improve neonatal outcomes worldwide.

### Technological innovations

The advances in technology have brought revolutionary changes in neonatal resuscitation, which includes improved ventilation, monitoring, and simulation-based training.

#### Monitoring advances

Technology has greatly influenced clinical SpO₂ monitoring and heart rate assessment guidelines. In the delivery room, the use of pulse oximetry now enables clinicians to obtain immediate SpO₂ outcomes, which is vital for the titration of oxygen supply. According to AAP and AHA guidelines, pulse oximetry is to be used in the first minutes of life for the guidance of oxygen therapy [34]. This has reduced the risks of hyperoxia and hypoxia, both being detrimental to newborns.

#### Ventilation techniques

Advancements in respiratory support devices have made ventilation safer and more effective. Innovations such as T-piece resuscitators, providing controlled positive pressure, have replaced self-inflating bags as they ensure uniform and accurate pressure delivery with the additional advantage of lower intubation rates [35]. These devices reduce barotrauma and improve lung recruitment, particularly in premature infants. Furthermore, non-invasive ventilation methods, such as CPAP and non-invasive positive pressure ventilation (NIPPV), have revolutionized the field by minimizing the risk of ventilator-associated lung injury. A respiratory function monitor (RFM) in the delivery room can help by adding objectivity to the clinical assessment in respiratory support training of healthcare personnel, and it has the potential for guiding and optimizing delivery room resuscitation. However, high cost, difficulty in interpretation during resuscitation, and lack of clinical evidence do not support the routine use of RFM. A recent systematic review including three RCTs (*n* = 443) did not find improvement in clinical outcomes like intubation rate, air leaks, mortality, severe IVH, and BPD with RFM use during PPV [36].

#### Simulation training

Advancements in technology have also improved neonatal resuscitation training with high-fidelity simulation programs. The NRP developed by the AAP incorporates simulation-based learning, improving skills, teamwork, and decision-making during resuscitation [5]. Engaging in simulation training improves the confidence of providers and lowers the likelihood of errors in real-world scenarios [37].

The application of new-age technologies has increased the levels of accuracy and reliability in neonatal resuscitation while making sure that healthcare workers are skilled in the application of evidence-based procedures.

#### Expert consensus and global collaboration

Collective inputs from pediatricians, neonatologists, and international organizations, along with researchers, have made a strong foundation for the development and evolution of neonatal resuscitation guidelines. The guidelines are globally admissible and will be continually updated with an evidence-based approach.

#### Role of professional organizations

The AHA, AAP, and ERC conduct critical activities in reviewing the evidence and creating standards. The ILCOR serves as a global platform for arriving at consensus on reviewing scientific information and developing treatment recommendations [6]. Thus, the annual updating of treatment advice guidelines by the AHA will improve ILCOR recommendations, ensuring that they are disseminated globally as the gold standard for neonatal resuscitation [2, 4].

#### Global health initiatives

The AHA and AAP guidelines and those developed by the WHO on basic newborn resuscitation for low-resource settings focus on low-cost interventions that contribute to the global reduction of neonatal mortality [17]. These guidelines include practice areas such as DCC, thermal care, and bag-and-mask ventilation techniques for simple resuscitation. With the collaboration of global health organizations strengthened, evidence-based resuscitation practices are now more accessible and disseminated to low- and middle-income countries.

#### Interdisciplinary efforts

Neonatal resuscitation involves a team of obstetricians, neonatologists, midwives, and nurses. The expert consensus highlights teamwork, communication, and a clear definition of duties in any resuscitation effort. For instance, training through NRP and Helping Babies Breathe® (HBB), and similar initiatives, intends to provide an effective and standardized approach towards newborn care across diverse clinical settings [20, 38].

#### Consensus building

It is the iterative process of developing the guidelines from a critical review of huge emerging evidence and the opinions of experts that contributes to real-world applicability. The relevance of current research and continued collaboration around the world has led to refinement of treatment protocols on neonatal resuscitation in the 2023 update of AHA and AAP [7, 39]. Global collaboration has facilitated sharing of knowledge and improving the implementation across different health systems, resulting in a decrease in neonatal mortality rates.

## Challenges and controversies

### Disagreements in practices

Neonatal resuscitation has always been a subject of debate, particularly for the best practice methods, ideal timing, and the most efficient technology. One such point of contention is the debate on DCC versus immediate clamping. Several studies, such as the one by Lu et al., have proven the benefits of DCC for improved outcomes, hemodynamic stability, and blood transfusion requirements in preterm infants [39]. Effective communication and teamwork cannot be ignored as deciding factors for neonatal resuscitation outcomes [40]. Some evidence has not been uniformly applied owing to the time estimated for resuscitation [6]. It is very challenging for most clinicians to balance the advantages of DCC with the urgent need for ventilation of critically ill neonates.

There is still controversy regarding the application of PPV. According to current guidelines, an infant should receive PPV within the first minute if the first round of stimulation attempts fails [10]. There is still disagreement regarding the use of a self-inflating bag, flow-inflating bag, or T-piece resuscitator. Studies that have used a T-piece resuscitator showed that it can achieve better pressure control; however, these findings are based on locations where access to this device is not feasible [25].

Further, the transition of preterm infants from invasive respiratory support to CPAP or other forms of non-invasive support continues to be a subject of debate. According to Manley et al. [22], non-invasive forms reduce the risk of ventilator-induced lung injury and bronchopulmonary dysplasia (BPD); however, these require sufficient training and facilities for successful implementation [26].

### Cultural and regional differences

Other aspects of culture and differences in the regional healthcare systems play a role in neonatal resuscitation practices. In some low-resource areas, cultural practices related to delivery and newborn care have been shown to cause delays in resuscitation. For example, reliance on traditional birth attendants is one such unique practice that prevents timely interventions for medical needs [17].

While North America and Europe remain the most advanced regions in adopting the current guidelines [2], other nations with fractured health systems may experience different results of resuscitation due to the lack of trained personnel in some areas. According to the findings of Kariuki et al., the recommendations of the ERC and AHA need to be aligned with healthcare services available in the specific region [25].

Moreover, medical treatment varies across different cultures; in some regions, the shortage of evidence-based insights on neonatal death has made it challenging to adopt evidence-based resuscitation practices [20]. Thus, it is necessary to provide culturally sensitive education and localized implementation strategies to address these differences.

### Implementing guidelines in low-resource settings

The most important challenge in neonatal resuscitation is the successful application of evidence-based guidelines in healthcare facilities with limited resources. Many guidelines have the assumption that the staff would make use of sophisticated equipment and trained personnel, which is often not true in the case of most underdeveloped regions. For example, Goldsmith and Martin affirm that access to PPV devices, pulse oximetry, and appropriate warming equipment is not available in many healthcare facilities, though it is essential [40].

Training deficits form another major bottleneck. Interventions such as the NRP have resulted in advances in establishing a standardized training program globally [5], but areas of low resources still experience a lack of sufficient trainers and sustained support. Simulation-based training has great potential in further improving skills; however, such methods may be impractical in facilities where infrastructure is limited [37].

There is also a lack of needed medications and disposables, such as oxygen, masks, and suction devices. In this scenario, simplified resuscitation tools and guidelines, like those recommended by WHO, provide a form of alternative intervention [15]. It cites basic procedure interventions as tactile stimulation, ventilation with room air, and thermal protection, which are applicable in resource-limited settings.

Overcoming all these hurdles would require collaborative efforts with global partnerships and innovative approaches. Successful improvements in neonatal outcomes have been achieved in several low-resource settings by assisting newborns’ breathing through simple, cost-effective interventions applied by trained professionals [38]. Further progress calls for investment in healthcare systems, workforce development, and the promotion of educational initiatives within communities.

## Case studies and evidence from practice

### Case studies

Numerous instances illustrate the impact of implementing neonatal resuscitation guidelines in real-life contexts, particularly the introduction of DCC into neonatal management practices, especially for preterm newborns. The finding of a randomized clinical trial by Yao et al. demonstrates that DCC lowers mortality and severe intraventricular hemorrhage in preterm neonates [41]. A neonatal intensive care unit in the UK showed a 15% reduction in neonatal transfusion rates following the introduction of DCC as a standard procedure. The positive outcomes experienced by hospitals employing this practice led to its inclusion in the AHA recommendations issued in 2020 [2].

Early CPAP initiation has also been shown to be advantageous in preterm neonates. It was established that implementing protocols for non-invasive ventilation decreased intubation and BPD rates among neonates born at less than 30 weeks of gestation [42]. This case demonstrates that research evidence should be implemented in clinical practice to ensure better outcomes in neonates. Furthermore, it is recommended that every birth should be attended by a doctor or staff trained in neonatal resuscitation program for a better outcome [43].

The benefits of the implementation of recommended guidelines have not only demonstrated improved outcomes in high-resource areas but also in low-resource ones. In the rural regions of sub-Saharan Africa, the HBB initiative trained birth attendants with basic resuscitation techniques [17]. With HBB, neonatal mortality dropped to 47%, as reported in the increased survival rates post-HBB implementation [25]. This exemplifies how simple, realistic instructions aligned with available resources can result in significant benefits.

### Lessons learned from practice

Though lessons learned from the implementation of neonatal resuscitation guidelines were substantial, they also varied for several reasons. The foremost reason is that few situations adequately prepare one for effective multidisciplinary collaboration. Successful implementation typically involves robust integrated efforts of pediatricians, neonatologists, and nurses, with the support of healthcare administrators. As demonstrated by Wyckoff et al., good teamwork in resuscitation scenarios significantly improves outcomes when the roles are well-defined and clear, and effective communication is maintained [6].

Second, continuing education and training were introduced for the adoption of practices as per the guidelines. The NRP has been an important factor for the whole world to standardize the training for resuscitation, which is approved by the AAP; however, as found in resource-poor countries, all such educational initiatives need to be context-sensitive [5]. Simulation-based training appears to be effective, especially for retaining resuscitation skills [36].

Thirdly and importantly, real-world evidence has proved that close monitoring and continuous feedback promote improvement. Hospitals that adopted quality improvement initiatives laid down by the guidelines, such as data audits and debriefings after resuscitation, reported successful compliance and improved neonatal outcomes.

Finally, the continuous theme of adaptation of guidelines in local contexts has been an integral part of this exercise. While global guidelines lay the framework, regional adaptations become necessary due to resource constraints, cultural beliefs, and limitations of the healthcare system. Thus, in areas where there is poor access to advanced ventilation devices, the use of low-cost alternatives, like bag-and-mask ventilation, has been emphasized [8].

## Summary

Overall, it is true that neonatal resuscitation has undergone the most remarkable evidence-based journey; it has indeed changed the landscape of neonatal care to one that improves the chances of survival for newborns worldwide. By reviewing the evolution of such guidelines, it becomes evident how real-life experience has driven the rigorous research and how they have relied on the technology that has been created to this end. Among these are the implementation of DCC and UCM, preference for early CPAP over invasive ventilation, and simplified, resource-appropriate protocols for low-income regions. These advancements reflect a pediatric practice readily adaptable to respond to various clinical and contextual challenges around the world.

The impact of these guidelines tends to be extremely significant to the current practices. Delayed cord clamping, once a subject of debate, is now more widely seen as a standard practice that reduces mortality and improves hemodynamic stability, especially in preterm newborns. The adoption of noninvasive respiratory support strategies has reduced the incidence of complications associated with mechanical ventilation involving ventilator-induced lung injury and BPD. Application of aggressive compared with gentle resuscitation has reduced lung injury and mortality from BPD. Structured training avenues such as the NRP and initiatives such as HBB have successfully narrowed the differences in skill and resource availability so that proper resuscitation practice can extend to different regions.

Although the advancement in knowledge and application of the best evidence has been substantial, it still raises challenges, such as existing regional differences in caregiving, lack of resources in lower income places, and sometimes debates on ventilation device selection and ideal oxygenation methods. These gaps need to be addressed to bring convergent results for all. New clinical trials and technological innovations will most likely continue to drive future changes in neonatal resuscitation guidelines. Some areas of investigation include the optimization of DCC timing and duration, efficacy and long-term safety of UCM in preterm infants, the best strategy for oxygen titration to minimize oxidative stress, and evidence for the benefit of the application of sustained lung inflation techniques. Further, advances in telemedicine, artificial intelligence, and simulation-based training offer a promising future for resuscitation, even more so in resource-limited settings.

## Conclusion

In conclusion, guidelines on neonatal resuscitation have presumably made significant progress toward improving care over the years, and much remains to be done through research and newer technological advances that will help close other gaps and promote safe approaches to future clinical needs. The emerging future promises to advance neonatal resuscitation practices through prioritizing equity, extensive training, and the use of cutting-edge technology, thereby leading to higher neonatal survival rates and providing the best possible start to every infant’s life.

## Data Availability

No datasets were generated or analysed during the current study.
